# Psychopathological Profile of Patients with Moderate-to-Severe Plaque Psoriasis and Its Correlation to DLQI: Results from a Prospective, Monocentric Clinical Study

**DOI:** 10.3390/jcm13216424

**Published:** 2024-10-26

**Authors:** Natalia Rompoti, Sofia Tsiori, Konstantinos Kontoangelos, Anastasios Kouzoupis, Charalabos Papageorgiou, Stamatios Gregoriou, Alexander Stratigos, Dimitrios Rigopoulos

**Affiliations:** 1 1st Department of Dermatology-Venereology, Andreas Sygros Hospital, Medical School, National & Kapodistrian University of Athens, 16121 Athens, Greece; natalia.rompoti@gmail.com (N.R.); alstrat2@gmail.com (A.S.); dimitrisrigopoulos54@gmail.com (D.R.); 2 1st Department of Psychiatry, Eginition Hospital, Medical School, National & Kapodistrian University of Athens, 11528 Athens, Greece; sofiatsiori@gmail.com (S.T.); kontoangel@med.uoa.gr (K.K.); akouzoup@med.uoa.gr (A.K.); chpapag@med.uoa.gr (C.P.); 3 Neurosciences and Precision Medicine Research Institute “Costas Stefanis”, University Mental Health, 15601 Athens, Greece

**Keywords:** psychopathology in psoriasis, alexithymia, DLQI, depression, anxiety

## Abstract

**Background**: Psoriasis is associated with a high psychological burden and comorbidities, such as depression and anxiety. The aim of this study was to evaluate the psychopathological profile of patients with moderate-to-severe plaque psoriasis under systemic treatment and to explore the association between DLQI and alexithymia, depression, and other psychopathological disorders. **Methods**: In this monocentric, prospective clinical study, 104 adult patients with moderate-to-severe plaque psoriasis were evaluated according to the disease severity (measured by PASI) influence of psoriasis on their quality of life (measured by DLQI) and their psychopathological profile (measured by the BDI, TAS-20, and SCL-90 questionnaires). **Results**: The psoriasis patients exhibited high levels of psychopathological symptoms, particularly for depression, obsessive-compulsive behavior, somatization, interpersonal sensitivity, and anxiety. More than half of the psoriatic patients (56.7%) were diagnosed with minimal depression, 26.9% with mild depression, and 16.3% with moderate or severe depression. The symptoms of possible and confirmed alexithymia were present in 19.2% and 15.4% of the patients, respectively. The patients with alexithymia appeared to experience a more significant impact on their QoL. Specifically, the percentage of patients with alexithymia/possible alexithymia who had a DLQI of ≥2 was 77.8% vs. 51.5% in those without alexithymia. **Conclusions**: Our study illuminates the intricate connection between the disease severity and psychological components that impact the QoL of patients with moderate-to-severe plaque psoriasis. It is advised that clinicians adopt a comprehensive approach to managing psoriasis, which involves addressing both the physical symptoms of the condition and the psychological impact. In cases of patients with a DLQI of ≥2, despite adequate clinical responses, evaluating the possible coexistence of general psychopathology is recommended.

## 1. Introduction

Chronic plaque psoriasis is a relapsing, inflammatory skin disease that impacts a significant percentage (0.1–11.4%) of the world’s population [1]. Characterized by visible erythematous and scaly and occasionally itchy plaques, psoriasis is associated with both significant physical discomfort and psychological distress, leading to impairments in patients’ quality of life (QoL) [1,2,3,4,5]. This translates into a cumulative, wide-ranging life course impairment that extends beyond physical symptoms, affecting different aspects of daily life such as productivity at work, interpersonal relationships, and psychological well-being [5].

It is well known that patients with psoriasis frequently encounter interpersonal challenges, challenges pertaining to one’s physical appearance, low self-worth, and low self-perception. Because of their appearance, they frequently feel stigmatized and embarrassed. This is frequently linked to the notion that others evaluate them based on their body image [6]. In order to avoid social implications, people with psoriasis usually develop coping mechanisms. A majority of these strategies, though, are ineffective in enhancing the patients’ quality of life [7,8]. There is strong evidence that discussing their skin condition with others, covering up afflicted areas of their body, and avoiding social interactions all negatively impact their quality of life. Discussing the non-contagious nature of psoriasis with others can reduce this negative impact and, as a result, social distress [9]. In an effort to objectify this QoL impairment, clinicians have widely used the Dermatology Life Quality Index (DLQI) [10].

Numerous studies have highlighted an association between psoriasis severity, as assessed by reliable clinical tools, including the Psoriasis Area and Severity Index (PASI), and decreased QoL, which is reflected by a high DLQI score [11]. This emphasizes the substantial influence of the disease severity on the subjective well-being of patients and underlines the significance of implementing suitable treatments to alleviate both the physical and psychological burdens of psoriasis.

In addition to the severity of the skin lesions, psychological variables can have an independent impact on the quality of life outcomes in psoriatic patients. For instance, they frequently exhibit depression and psychopathology symptoms, which are associated with poorer QoL outcomes [12]. Feelings of shame, humiliation, and social stigma are among the psychological consequences of psoriasis that can worsen pre-existing psychiatric symptoms and contribute to heightened psychological distress [13]. It is essential to focus on treating depression and psychopathology in order to enhance the QoL and overall well-being of psoriatic patients.

Moreover, alexithymia, a personality trait defined by difficulties in identifying and expressing emotions, has been linked as a factor that contributes to reduced QoL in individuals with psoriasis [13,14]. People with alexithymia may find it difficult to deal with the emotional difficulties associated with their disease, which can result in increased distress. It is crucial to comprehend the connection between alexithymia and DLQI in psoriatic patients in order to create specific interventions that target emotional well-being in this population.

The aims of this study were (1) to evaluate the psychopathological profile of patients with moderate-to-severe plaque psoriasis under systemic treatment, (2) to explore the association between alexithymia and DLQI, and (3) to assess the predictive value of various factors, including PASI, alexithymia, depression, and psychopathology, on DLQI in psoriatic patients.

## 2. Materials and Methods

### 2.1. Sample and Procedure

In this prospective, single-center clinical study, 104 adult patients with moderate-to severe, chronic (>6 months) plaque psoriasis under systemic treatment for at least 16 weeks with either conventional drugs or small molecule or biologic agents were recruited between April 2018 and March 2020 from the Psoriasis Department of our tertiary hospital. Demographic and clinical data were collected in all cases. PASI and DLQI were used to assess the severity of psoriasis and the disease’s impact on QoL, respectively. PASI was assessed at the initiation of the present systemic therapy and subsequently re-evaluated at the timepoint of inclusion to this clinical study to evaluate response to treatment. In order to assess the psychopathological profiles of the patients, the Toronto Alexithymia Scale (TAS-20), Beck Depression Inventory (BDI), and Brief Symptom Inventory SCL-90 (SCL-90) were administered at the study’s initiation. Ethical approval was obtained from the Institutional Review Board. This study excluded patients with learning disabilities and difficulties with illiteracy.

### 2.2. Assessment Instruments

PASI, ranging from 0–72, is the most widely accepted and frequently used index to assess skin psoriasis severity. The erythema, scaling, and plaque thickness are the three distinct factors that this index uses to assess the severity of psoriasis. Additionally, it evaluates the percentage of the head, trunk, and upper and lower extremities that are affected by the disease. When plaque psoriasis has a PASI score of ≥10, it is commonly classified as moderate-to-severe, with a score of ≥10 being indicative of moderate-to-severe disease [15].

DLQI, ranging from 0–30, was used to assess QoL. A higher DLQI score means greater impairment in the patient’s QoL. The DLQI is a simplified, self-administered questionnaire that takes 126 s, on average, to complete. It consists of ten questions asking patients to rate how various aspects of their quality of life have been affected by skin conditions during the past week. Validation has been achieved for dermatology patients aged sixteen years and above. The DLQI’s items cover a wide range of topics, including feelings and symptoms, everyday activities, leisure, career or school, interpersonal relationships, and treatment side effects. Each item is scored on a four-point Likert scale as follows: 0, not at all ⁄not relevant; 1, a little; 2, a lot; and 3, very much. Scores for individual items (0–3) are added to yield a total score (0–30), and higher scores mean greater impairment in the patient’s QoL. Specifically, 0–1 indicates no impact on the patient’s life, 2–5 indicates a minor impact, 6–10 indicates a moderate/intermediate impact, 11–20 denotes a significant impact, and 21–30 denotes an extremely significant impact on the patient’s QoL [10,16].

The Beck Depression Inventory (BDI) is a widely utilized 21-item self-report instrument designed to evaluate the severity of depression. The updated version encompasses a range of indicators related to the emotional, cognitive, and physical aspects of depression. These include emotional experiences such as hopelessness and irritability, cognitive distortions like guilt or perceptions of punishment, and somatic symptoms such as fatigue, weight loss, and diminished sexual interest. The standard cut-offs are as follows: 1–9 indicates minimal, 10–18 indicates mild, 19–29 indicates moderate, and 30–63 indicates severe depression [17].

The Toronto Alexithymia Scale (TAS-20) is a 20-item self-report tool that assesses alexithymia, with responses rated on a 5-point Likert scale ranging from 1 (strongly disagree) to 5 (strongly agree). The total score (TTL) can range from 0 to 100, and the scale is divided into the following three subscales: difficulty identifying feelings (DIF), difficulty describing feelings (DDF), and externally oriented thinking (EOT). According to the accepted standard, a total score exceeding 61 is indicative of alexithymia, a score of 52–60 suggests intermediate/borderline alexithymia, and a score of <51 indicates the absence of alexithymia. The TAS-20 has been translated and validated in Greek [18].

Nine dimensions of psychopathology are measured by the Symptom Checklist-90 (SCL-90), a self-report questionnaire, as follows: (1) somatization, (2) depression, (3) anxiety, (4) phobic anxiety, (5) obsessive-compulsive behavior, (6) paranoid ideation, (7) psychoticism, (8) hostility, and (9) interpersonal sensitivity. With 90 items total, each question is scored from 0 to 4, with a maximum possible score of 360. The following three global indices are also provided by the scale: the Positive Symptom Total, the Positive Symptom Distress Index, and the General Severity Index. A validated Greek version of the scale is available for use [19].

### 2.3. Statistical Analysis

Variables with normal distribution are presented descriptively with means and standard deviations, while variables for which normal distribution was not confirmed are presented with medians and interquartile ranges. Categorical variables are presented with frequencies, relative frequencies, and bar graphs.

The Mann–Whitney test was applied to examine the relationship of the psychopathology scales with the categorical demographic characteristics, clinical findings, and severity of psoriasis. The Spearman correlation coefficient was applied to examine the relationship of the clinical characteristics, as continuous variables, with the psychopathology scales. The Spearman correlation coefficient was used to examine the relationship of alexithymia with the psychopathology scales.

The relationship of DLQI (used as a dichotomous variable) with demographic characteristics, clinical findings, PASI, and alexithymia were examined, and the Mann–Whitney test was applied when the variables were continuous while the χ^2^ test was used when the variables were categorical.

All the models presented were weighted for gender and age, and the forward variable selection method was applied. The level of statistical significance was set at 0.05. The statistical analysis was performed with the SPSS program (Statistical Package for the Social Sciences, IBM SPSS Statistics for Windows, Version 24.0).

## 3. Results

The results of the PASI are presented in Table 1. Although all patients were diagnosed with moderate-to-severe plaque psoriasis, 61.1% had a PASI score of <10 at systemic treatment initiation (mainly due to a direct switch from a previous inadequate treatment), 26.3% had a PASI score of 10–15, and 12.6% had a score of >15. At the time of study initiation and for those who had been under systemic treatment for at least 16 weeks, 59.6% of the cohort had a PASI score of ≤1, 78.8% had a score of ≤2, 89.4% had a score of ≤3, and 95.2% had a score of ≤5. A comparison of the PASI differences between the two assessments revealed that 71.6% of the patients experienced a reduction of up to 75% (PASI score of 75), 52.6% experienced a reduction of up to 90% (PASI score of 90), and 36.8% experienced complete clearance of the skin, clinically (100% reduction in baseline PASI, with a PASI score of 100).

Regarding DLQI under treatment at the time of study initiation and psychopathological evaluation, our data showed that in 39.4% of the patients, there was no effect (DLQI 0/1); in 35.6% of the patients, there was little effect; in 19.2% of patients, there was an intermediate effect; in 4.8% of patients, the effect was high; and in 1.0% of patients, there was a very high effect of the psoriasis on their QoL.

Concerning BDI depression scale, our data showed that more than half of psoriatic patients (56.7%) were diagnosed with minimal depression, 26.9% with mild depression, and 16.3% with moderate or severe depression. In our sample, 4.0% of the patients had a history of psychiatric disorders prior to the study. Specifically, 3.0% of the sample had been diagnosed with depression and were under antidepressant treatment at the time of the study.

The alexithymia levels of the psoriatic patients assessed by the TAS-20 showed no symptoms of alexithymia in 65.4% of the sample, though there were symptoms of possible alexithymia in 19.2% and confirmed alexithymia in 15.4%.

The SCL-90 assessment revealed that the psoriasis patients exhibited high levels of psychopathological symptoms, particularly for depression, obsessive-compulsive behavior, somatization, interpersonal sensitivity, and anxiety. The high values in these subscales were derived from comparing the quartile distributions of each subscale.

### Demographic Characteristics and Clinical Findings of the Psoriatic Patients in Relation to Their Quality of Life

The demographic characteristics and clinical findings of the patients in relation to the DLQI were assessed, in particular, we compared the patients in whom the disease had no effect compared to those in whom at least minor effects were evaluated (Table 2). The percentage of women who experienced at least a slight impact on their quality of life was statistically significantly higher than that for men (74.4% versus 50.8%, respectively; *p* = 0.015). In the subgroup of patients with psoriatic arthritis (PsA), 65.5% had a DLQI of ≥2 and 34.5% had a DLQI of 0/1. Among the systemic treatments that have been associated with mood disorders based on approved clinical studies, 9.6% of the patients were being treated with brodalumab and 19.2% with apremilast. Regarding the other patients, 5.8% were receiving treatment with acitretin, 5.8% with adalimumab, 1.9% with certolizumab pegol, 5.8% with cyclosporine (CYA), 6.7% with Enstilar, 5.8% with etanercept, 1.0% with infliximab, 5.8% with methotrexate (MTX), 16.3% with secukinumab, and, finally, 17.3% with ustekinumab. No statistically significant relationships were observed with respect to the other demographic characteristics and clinical findings of patients and the impact of the disease on their QoL.

Concerning PASI in relation to DLQI, there was no statistically significant relationship between the DLQI and initial PASI values or the values for those under treatment at the time of psychopathological evaluation (Table 3). However, the patients who experienced clinical improvements of 75% appeared to have less affected QoL. Specifically, 52.9% vs. 81.5% who reached PASI75 or not, respectively, had a small or greater disease-related impact on their QoL (*p* = 0.010).

The degree of alexithymia among the psoriatic patients was also examined for its correlation with DLQI (Table 4). The patients whose QoL (measured by DLQI) was affected, even to a minor extent, exhibited greater difficulty in recognizing their emotions compared to those whose quality of life remained unaffected (17.0 vs. 11.0, respectively; *p* = 0.039). Additionally, the patients with alexithymia appeared to experience a more significant impact on their QoL than those without alexithymia. Specifically, the percentage of patients with alexithymia/possible alexithymia who had a DLQI score of ≥2 was 77.8% compared to 51.5% in those without alexithymia (*p* = 0.009).

In order to evaluate the effect of treatment on the psychopathological profile and QoL of this patient collective, logistic regression models were performed (Table 5). The psoriatic patients who did not achieve PASI75 were four times more likely to have their QoL negatively impacted compared to those who achieved at least a 75% improvement in their initial PASI score (OR = 4.2, *p* = 0.012), given the same levels of alexithymia. The patients with alexithymia were 2.5 times more likely to experience a negative impact on their QoL from the disease compared to the patients without alexithymia, independent of PASI response. However, this difference was statistically significant only at the 10% level (OR = 2.5, *p* = 0.080). Subsequently, the relationship between PASI75 and DLQI was examined, taking into account gender, age, BDI scale, and each scale of the SCL90. The psoriatic patients who did not achieve PASI75 were 4.4 to 4.9 times more likely to have a DLQI score of ≥2 compared to those who did, assuming they were of the same age and gender and had the same levels on the BDI scale or each SCL90 scale. If the value on the BDI scale or any of the SCL90 scales, except for phobic anxiety or the psychopathology index, increased by one unit, the likelihood of the disease negatively impacting the QoL increased by 10% or 20%, assuming that age, gender, and response to treatment (PASI75) remained constant.

## 4. Discussion

Our study highlights the detrimental effects of psoriasis on QoL for a majority of patients, which is in line with former research data [20,21,22]. Multiple reasons have been linked to the negative impact, including patients’ financial difficulties, mental health problems, and interpersonal relationship issues [8,23,24]. In a previous paper, Arancio et al. (2022) [25] highlighted that approximately 80–90% of psoriasis patients say they have encountered stigmatization and discrimination because of their condition, which has a negative effect on their personal and professional lives. Up to 20% of these people have experienced difficulties getting into public areas such as gyms and swimming pools. Frequent discrimination like this can result in low self-esteem and missed opportunities for meaningful relationships, career advancement, and personal fulfilment. Furthermore, the social disengagement brought on by stigmatization may make them more vulnerable to psychological disorders and raise their chances of developing addictive behaviors like substance abuse, alcoholism, and smoking and food addiction [25,26].

In the present study, the percentage of women who experienced a considerably greater influence on their QoL was significantly greater than that for men. Previous data have also underlined the gender differences concerning the impact of this skin disease on patients’ QoL, as measured by the DLQI [27]. Psoriasis provokes emotions of shame, frustration, and irritation among female patients. Due to the visible changes in their skin, some female patients perceive themselves as less attractive, leading to social withdrawal and reduced interpersonal engagement. This could be attributed to the widespread acceptance of the assumption that women are more dependent on interpersonal relationships and more concerned with their physical appearance compared to men. These findings suggest that when assessing the severity of psoriasis in females, measuring QoL, for instance, via DLQI is of particular importance [28,29].

A majority of the patients with PsA had DLQI scores of ≥2. Assessment of the subpopulation with DLQI scores of ≥2 revealed that 65.5% vs. 58.1% were with or without joint involvement, respectively (arithmetical but not statistical significance). Systematic reviews with meta-analyses of patients with PsA have revealed that 11.9–20% suffered from depression, 19–33% suffered from anxiety, and 38% suffered from alexithymia, which could all have contributed to the higher DLQI scores [23].

Interestingly, in our cohort, there was no significant correlation between the absolute PASI for before and under treatment and the DLQI-measured QoL. Nevertheless, the patients who achieved a clinical improvement of at least 75% exhibited a substantial difference in their DLQI. This could be attributed to the fact that this study included only patients with moderate-to-severe plaque psoriasis, and a majority of the patients with PASI scores of <10 had experienced a switch in their treatment at study initiation. Thus, these data indicated that the QoL impairment due to psoriasis was received differently and independently from the visible psoriatic plaques in each patient. Moreover, even a mild-to-moderate lesion recurrence under treatment (PASI score of <10) could have a great impact on a psoriatic patient. However, when the patients achieved at least a 75% clinical improvement in their PASI, the skin disease had a lower impact on their QoL. This highlights the importance of focusing on significant therapeutic improvements in the treatment of psoriasis to improve the overall well-being of patients. Previous studies have also suggested that attaining at least PASI75 would signify improved management of psoriasis symptoms, resulting in reduced physical discomfort, pain, and irritation caused by psoriasis lesions [30,31], as well as psychological distress. Furthermore, in a previous study, it was highlighted that patients with dermatological conditions frequently exhibit a distorted self-image which can affect their quality of life. Specifically, if pathological lesions cover a significant area of an individual’s skin, especially exposed areas like the face, palms, and external genitals, they feel stigmatized or even “disgusting”. These feelings have a detrimental impact on the psychological state of dermatological patients, as well as on their social, professional, and family environments. They also lead to a significant decline in their quality of life [32,33]. In addition, one of the most significant factors influencing the severity of psoriasis is itching, which is also one of the most bothersome symptoms of the condition. Prior randomized, controlled trials (RCTs) have demonstrated that the HRQoL of psoriasis patients is significantly enhanced by improvements in itching and PASI response [34,35]. Therefore, achieving at least PASI75 indicates effective disease management and control of symptoms in these patients, resulting in physical, psychological, and social advantages that enhance their overall QoL [36,37,38].

Extensive research has been conducted on the correlation between alexithymia and QoL in patients with psoriasis. Prior studies have shown that alexithymia in this population is associated with difficulties in both recognizing and expressing emotions [39,40]. This can result in difficulties with coping with stressors and effectively managing chronic diseases like psoriasis [40,41]. In addition, there seems to be an impact of alexithymia on the exacerbation of the psychological distress caused by psoriasis [6,42]. This results in more significant limitations in social relationships, as well as reduced compliance with treatment. Our findings also suggest that patients who experience any degree of impairment in their QoL due to psoriasis tend to have greater difficulty in recognizing their emotions than those whose QoL remains unaffected. Furthermore, those with alexithymia experience a greater impact of psoriasis on their QoL, highlighting the importance of emotional recognition in managing the psychological and social impacts of the condition. Specifically, our data revealed that patients with alexithymia were 2.5 times more likely to experience a negative impact on their QoL (DLQI score of ≥2) from the disease compared to patients without alexithymia, independent of PASI response. Thus, conducting comprehensive assessments and implementing customized interventions that focus on improving emotional awareness and coping skills is important. This could enhance overall well-being and should be part of psoriasis management, especially in cases where PASI75 had been reached and DLQI scores reveal continued impairment in patients’ QoL (DLQI ≥ 2).

Our research has also confirmed that depression and psychopathology are also variables which can predict an impaired QoL. Increased levels of depression and psychopathological symptoms, including anxiety disorder, have been associated with a higher likelihood of encountering negative QoL consequences. This is in line with earlier research [11,12,43]. Common pathogenetic pathways between psoriasis and depression, with the overproduction of cytokines such as IL-6, IL-17, and TNFa, could contribute to this phenomenon [44,45].

Analyzing the psychopathological profiles of psoriatic patients revealed that obsessive-compulsive disorder is one of the most common disorders in this cohort. This has also been reported in other studies [46,47]. Furthermore, psoriasis patients exhibit higher levels of hypochondriasis and other aspects of somatization [48]. Psychosomatic factors, including attachment instability, a lack of social support, and traumatic life experiences, may also account for the elevated somatization rates observed in psoriasis patients [49]. In addition, psoriatic patients frequently experience feelings of shame and inadequacy as a result of their condition. These emotions lead to substantial social withdrawal and life disruption. Higher rates of interpersonal sensitivity may be attributed to the fear of rejection and criticism, which can result in feelings of personal inadequacy [50,51]. Thus, interventions that focus on improving emotional regulation skills are of great importance.

These findings emphasize the necessity of managing psychological discomfort since emotional well-being has a significant impact on the overall QoL of those affected with plaque psoriasis. Although the focus of our study was on the psychological burden and quality of life of patients receiving systemic treatment for moderate-to-severe plaque psoriasis, it is important to note that similar challenges are seen in various psoriasis subtypes. A previous study described treatment strategies for palmoplantar psoriasis (PPP) that highlight the importance of carefully taking into account comorbidities like depression and chronic infections, which are also relevant in managing patients with plaque psoriasis [52].

## 5. Limitations

Although prospective, this was a monocentric study conducted on 104 patients, and not all clinical data were available. Several limitations of our study should be acknowledged. First, the absence of patients with mild psoriasis and those treated only with topical medications may limit the generalizability of our findings to the broader psoriasis population. Additionally, we did not have access to DLQI, BDI, or SCL-90 scores prior to treatment initiation, which could have provided valuable insights into the baseline psychological statuses of the patients. Furthermore, the lack of data regarding psychiatric manifestations before psoriasis onset prevented us from exploring the potential bidirectional relationship between psychiatric comorbidities and the onset of psoriasis. Future studies should consider including these patient populations and collecting baseline psychological data to provide a more comprehensive analysis.

## 6. Conclusions

Our study illuminates the intricate connection between the severity of the disease and the psychological components that impact the QoL of patients with moderate-to-severe plaque psoriasis. We have pinpointed these factors in our research, which can be utilized to design personalized treatments aimed at improving the well-being of psoriasis patients. For instance, in cases where PASI75 has been reached but DLQI scores are ≥2, clinicians should perform the necessary steps to diagnose any underlying psychopathology, including alexithymia or depression. Thus, we recommend that clinicians employ a holistic strategy in the management of psoriasis, including treatment of both the physical symptoms and the psychological impacts of the condition. In order to improve patients’ QoL, it is essential to combine traditional medical therapies with a comprehensive psychological assessment and, if needed, intervention. This may involve therapies targeted at addressing alexithymia, depression, and psychopathology.

## Figures and Tables

**Table 1:** Psoriasis Area Severity Index (PASI) scores of psoriasis patients.

	Total Nr.	Nr. with No Missing Data
	*n* = 104	*n* = 89
PASI at treatment start, median (IQR)	7.20 (9.0)	7.20 (8.95)
≤10, *n* (%)	58 (61.1%)	55 (61.8%)
10–15, *n* (%)	25 (26.3%)	23 (25.8%)
>15, *n* (%)	12 (12.6%)	11 (12.4%)
PASI at study initiation under treatment, median (IQR)	0.60 (1.80)	0.60 (1.80)
≤1, *n* (%)	62 (59.6%)	53 (59.6%)
≤2, *n* (%)	82 (78.8%)	69 (77.5%)
≤3, *n* (%)	93 (89.4%)	80 (89.9%)
≤5, *n* (%)	99 (95.2%)	84 (94.4%)
Percentage of PASI improvement		
PASI 75, *n* (%)	68 (71.6%)	63 (70.8%)
PASI 90, *n* (%)	50 (52.6%)	47 (52.8%)
PASI 100, *n* (%)	35 (36.8%)	34 (38.2%)

**Table 2:** Demographic characteristics and clinical findings of the patients in relation to DLQI.

	DLQI		
	No Effect (DLQI 0–1)	At Least Minor Effect (DLQI ≥ 2)	*p*-Value
	*n* = 41 (39.4%)	*n* = 63 (60.6%)	
Age, median (IQR) (*n* = 104)	56 (14.3)	51 (17.0)	0.305
Gender, *n* (%)			
Male (*n* = 61)	30 (49.2%)	31 (50.8%)	**0.015**
Female (*n* = 43)	11 (25.6%)	32 (74.4%)	-
BMI (kg/m^2^), median (IQR)	21.8 (3.8)	22.8 (2.4)	0.237
Smoking, *n* (%)			
No (*n* = 38)	13 (34.2%)	25 (65.8%)	0.342
Yes (active or past) (*n* = 64)	28 (43.8%)	36 (56.3%)	-
Regular alcohol consumed, *n* (%)			
No (*n* = 58)	20 (34.5%)	38 (65.5%)	0.210
Yes (*n* = 45)	21 (46.7%)	24 (53.3%)	-
Comorbidities, *n* (%)			
No (*n* = 45)	20 (44.4%)	25 (55.6%)	0.480
Yes (*n* = 56)	21 (37.5%)	35 (62.5%)	-
Cardiovascular disease, *n* (%)			
No (*n* = 78)	28 (35.9%)	50 (64.1%)	0.077
Yes (*n* = 23)	13 (56.5%)	10 (43.5%)	
Metabolic disease, *n* (%)			
No (*n* = 73)	32 (43.8%)	41 (56.2%)	0.284
Yes (*n* = 28)	9 (32.1%)	19 (67.9%)	-
Age at PsO onset (years), median (IQR)	27.0 (19.8)	28.0 (20.0)	0.771
Age at treatment start (years), median (IQR)	51.0 (17.3)	48.0 (16.0)	0.560
Disease duration prior to treatment start (years), median (IQR)	15.0 (23.0)	17.0 (15.0)	0.776
Psoriatic arthritis, *n* (%)			
No (*n* = 74)	31 (41.9%)	43 (58.1%)	0.490
Yes (*n* = 29)	10 (34.5%)	19 (65.5%)	-

IQR, interquartile range. Results with statistical significance are presented in bold to facilitate easier identification of key findings.

**Table 3:** DLQI assessment in relation to PASI in this cohort.

	DLQI		
	No Effect (DLQI 0–1)	At Least Minor Effect (DLQI ≥ 2)	*p*-Value
	*n* = 41 (39.4%)	*n* = 63 (60.6%)	
PASI at treatment start, median (IQR)	7.2 (9.0)	7.6 (8.9)	0.380
PASI at study initiation, *n* (%)			
≤1 (*n* = 62)	26 (41.9%)	36 (58.1%)	0.524
>1 (*n* = 42)	15 (35.7%)	27 (64.3%)	-
PASI 75, *n* (%)			
No (*n* = 27)	5 (18.5%)	22 (81.5%)	**0.010**
Yes (*n* = 68)	32 (47.1%)	36 (52.9%)	-
PASI 100, *n* (%)			
No (*n* = 60)	20 (33.3%)	40 (66.7%)	0.142
Yes (*n* = 35)	17 (48.6%)	18 (51.4%)	-

IQR, interquartile range. Results with statistical significance are presented in bold to facilitate easier identification of key findings.

**Table 4:** Alexithymia in relation to DLQI.

	DLQI		
	No Effect (DLQI 0–1)	At Least Minor Effect (DLQI ≥ 2)	*p*-Value
	*n* = 41 (39.4%)	*n* = 63 (60.6%)	
TAS20 DIF, median (IQR) (*n* = 104)	11.0 (9.5)	17.0 (14.3)	**0.039**
TAS20 DDF, median (IQR) (*n* = 103)	11.0 (7.0)	13.0 (7.0)	0.229
TAS20 EOT, median (IQR) (*n* = 104)	18.0 (7.0)	18.5 (7.5)	0.550
TAS20 TTL, median (IQR) (*n* = 104)	41.0 (18.0)	49.5 (22.3)	0.110
TAS 20, *n* (%)			
No alexithymia (*n* = 68)	33 (48.5%)	35 (51.5%)	**0** **.009**
Possible or definite alexithymia (*n* = 36)	8 (22.2%)	28 (77.8%)	-

DDF, difficulty in describing feelings; DIF, difficulty in identifying feelings; EOT, externally oriented thinking; ΤΤL, total score; IQR, interquartile range. Results with statistical significance are presented in bold to facilitate easier identification of key findings.

**Table 5:** Logistic regression model predicting the association between DLQI, PASI75, TAS20, BDI, and SCL90.

	OR	95% ΔΕ	*p*-Value
PASI75, no	4.2	1.4–13.0	**0.012**
TAS20 TTL (possible or definite alexithymia)	2.5	0.9–7.0	0.080
PASI75, no	4.9	1.5–15.9	**0.009**
BDI	1.1	1.0–1.2	**0.002**
PASI75, no	4.6	1.5–14.6	**0.009**
Somatization	1.1	1.0–1.2	**0.006**
PASI75, no	4.4	1.4–13.9	**0.011**
Obsessive compulsive behavior	1.1	1.0–1.2	**0.012**
PASI75, no	4.4	1.4–13.6	**0.010**
Interpersonal sensitivity	1.1	1.0–1.2	**0.049**
PASI75, no	4.6	1.5–14.5	**0.009**
Depression	1.1	1.0–1.1	**0.013**
PASI75, no	4.6	1.5–14.3	**0.009**
Anxiety	1.1	1.0–1.2	**0.041**
PASI75, no	4.5	1.4–14.5	**0.011**
Hostility	1.2	1.0–1.4	**0.012**
PASI75, no	4.2	1.4–12.7	**0.012**
Phobic anxiety	1.0	0.9–1.2	0.537
PASI75, no	4.5	1.4–13.8	**0.010**
Paranoid ideation	1.1	1.0–1.2	**0.033**
PASI75, no	4.6	1.5–14.4	**0** **.** **008**
Psychoticism	1.1	1.0–1.2	**0.043**

Τhe models were adjusted for age and sex. OR, odds ratio; ΤΤL, total score. Results with statistical significance are presented in bold to facilitate easier identification of key findings.

## Data Availability

Data are available from the corresponding author upon reasonable request.
